# Cervical Pessaries for the Prevention of Preterm Birth: A Systematic Review

**DOI:** 10.1155/2013/576723

**Published:** 2013-03-31

**Authors:** Sophie M. S. Liem, Mariëlle G. van Pampus, Ben Willem J. Mol, Dick J. Bekedam

**Affiliations:** ^1^Department of Obstetrics and Gynecology, Academic Medical Center, P.O. Box 22770, 1100 DE Amsterdam, The Netherlands; ^2^Department of Obstetrics and Gynecology, Onze Lieve Vrouwe Gasthuis, 1100 DE Amsterdam, The Netherlands

## Abstract

*Introduction*. Reduction of preterm birth is a major goal in obstetric care. We performed a systematic review of randomized controlled trials and cohort studies on the effectiveness of the cervical pessary to prevent preterm birth. *Methods*. We searched the electronic databases of MEDLINE and Embase from inception until April 2012 to identify studies investigating treatment with a cervical pessary to prevent preterm birth. We constructed two-by-two tables for delivery before 28, 34, and 37 weeks of gestation and calculated relative risks (RRs) with 95% confidence intervals. *Results*. The search revealed 103 potentially eligible abstracts of which six cohort studies and four randomized controlled trials (RCTs) investigated the effectiveness of the pessary. One RCT (*n* = 380) demonstrated a lower delivery rate prior to 34 weeks (RR 0.24; 95% CI 0.13–0.43) in the pessary group, while another RCT (*n* = 108) showed no positive effect of pessary for delivery before 34 weeks (RR 1.73; 95% CI 0.43–6.88). Two older quasi randomized studies and cohort studies indicated potential effect of the pessary. *Conclusions*. Available randomized and nonrandomized studies indicate potential effectiveness of a cervical pessary in the prevention of preterm birth. More randomized clinical trials are needed before this device can be used in clinical practice.

## 1. Introduction

Preterm birth (PTB) is the most common cause of perinatal morbidity and mortality; therefore, preventing PTB is one of the most important targets in the current obstetric care. Mechanical prevention of preterm birth was proposed six decades ago by the use of the Shirodkar and McDonald cerclage [1, 2]. The effectiveness of these interventions has been assessed in two Cochrane reviews. 

The first Cochrane review among women with risk factors for preterm delivery or a history of miscarriages pooled the result of four studies (*n* = 1035) and showed no significant reduction in PTB <37 weeks when using a cerclage (RR 0.88, 95% CI 0.76–1.03). Three studies (*n* = 388) reporting on delivery before 32 weeks of gestation were pooled and none showed a reduction in preterm birth <32 weeks due to the cerclage (RR 1.29, 95% CI 0.67–2.49) [3].

The second Cochrane review on cervical cerclage among women with a singleton pregnancy at high risk of PTB based on their history (e.g., previous PTB, cervical surgery, short CL on ultrasound, or detected cervical changes) showed a significant reduction in PTB before 37 weeks of gestation (*n* = 2898, RR 0.80, 95% CI 0.69–0.95) and before 34 weeks of gestation (*n* = 2392, RR 0.79, 95% CI 0.68–0.93) [4]. In both reviews, cervical cerclage was associated with a higher rate of maternal side effects (pyrexia, vaginal discharge and bleeding) and larger number of caesarean sections. 

Meta-analysis assessing of the effectiveness of a cervical cerclage in women with a multiple pregnancy showed an increased risk of PTB before 35 weeks (RR 2.2, 95% CI 1.2–4.0) and a trend towards higher perinatal mortality (RR 2.7, CI 95% 0.83–8.5) [5].

The data discussed previously does not allow a firm conclusion on the use of cerclage to prevent preterm birth. While it should not be used in twins, at present there remains controversy on its effectiveness in singleton pregnancies.

An alternative for a cerclage is a cervical pessary. Vaginal pessaries have been used to prevent preterm birth since 1959 [6]. During the pregnancy the cervix normally stays tightly closed with a cervical mucus plug (CMP) sealing the opening. It is hypothesized that impairment of the CMP, for example, by cervical effacement, can lead to an ascending infection and preterm delivery; nevertheless, this needs further clarification [7]. The vaginal pessary encompasses the cervix and compresses the cervical canal, and so may prevent deterioration of the CMP. The pessary alternates the inclination of the cervical canal and corrects the incompetent cervix pointing forward in the axis of the vagina. It relieves direct pressure on the internal cervical os by distributing the weight of the pregnant uterus onto the vaginal floor, retrosymphyseal osteomuscular structures, and Douglas cavity and so may prevent premature dilatation of the cervix and premature rupture of the membranes. Furthermore, it blocks the fetal head from descending and pressing on the internal ostium [6, 8].

The cervical pessary is a relatively noninvasive, not operator-dependent intervention, which can be easily placed or removed in an outpatient clinic and does not require anesthesia. With speculum examination, the cervix is identified to determine an appropriate pessary size. The silicon Arabin pessary is the most popular and has different sizes of diameter and height. It is flexible and fits high around the cervix, so that the smaller inner diameter encompasses the cervix. After placement the patient is briefly observed to ensure there is no discomfort, vaginal blood loss, or uterine activity. 

The aim of this study is to systematically review the literature about the use of the cervical pessary to prevent preterm birth before 28, 34, and 37 weeks of gestation. 

## 2. Methods

### 2.1. Search Strategy

We searched the electronic databases of MEDLINE (US National Library of Medicine, Betheshda, MD, USA) and Embase (Elsevier, Amsterdam, The Netherlands) from inception to November 2012. The search was assisted by a clinical librarian and included “Obstetric Labor” AND “Premature" OR “Premature” OR “Preterm” AND “Birth” OR “Deliver” OR “Labor” OR “Labour” OR “Uterine cervical incompetence” OR “Cervix” OR “Cervical” AND “Incompetence” OR “Incompetent” AND “Pessaries” OR “Pessaries” OR “Pessary” MeSH or key terms. We checked reference lists to identify articles not found by electronic searches. We identified randomized controlled trials as well as cohort studies on the effectiveness of a cervical pessary to prevent preterm birth. 

### 2.2. Study Selection

Identified articles were screened by two independent reviewers (S. Liem and M. van Pampus) on title and abstract to determine their appropriateness for inclusion. The studies should have preterm birth as their primary or secondary outcome and report on the use of a cervical pessary. If studies could not be excluded based on their abstract or title, a full manuscript was obtained. We did not apply any language restrictions. If an article was written in a language other than Dutch or English, it was translated by a colleague with expertise in this language. If information available from the publications was not sufficient, the primary authors were contacted. If any disagreements about study inclusion arose, the two reviewers had a discussion. If consensus could not be reached a third reviewer (B. Mol) determined whether the study should be included. 

### 2.3. Data Extraction and Synthesis

The two reviewers abstracted the data separately. The following data and information were extracted from each eligible study: first author, year of publication, country, number of women, inclusion and exclusion criteria, type of pessary, definition of preterm birth, population demographics, neonatal outcome, pessary removal, and side effects. 

Methodological quality of included studies was determined by using the Delphi list for quality assessment of randomized clinical trials by both reviewers independently [9]. We evaluated the following items: treatment allocation, method of randomization, group's similarity at baseline, specified eligibility criteria, blinding of outcome assessor, blinding of care provider, blinding of patient, point estimates and measures of variability presented for the primary outcome measures, and intention-to-treat analysis. For the included studies, relative risks (RRs) and 95% confidence intervals (CIs) for delivery before 28, 34 and, 37 weeks of gestation were calculated from two-by-two tables. 

## 3. Results

**Figure 1:**
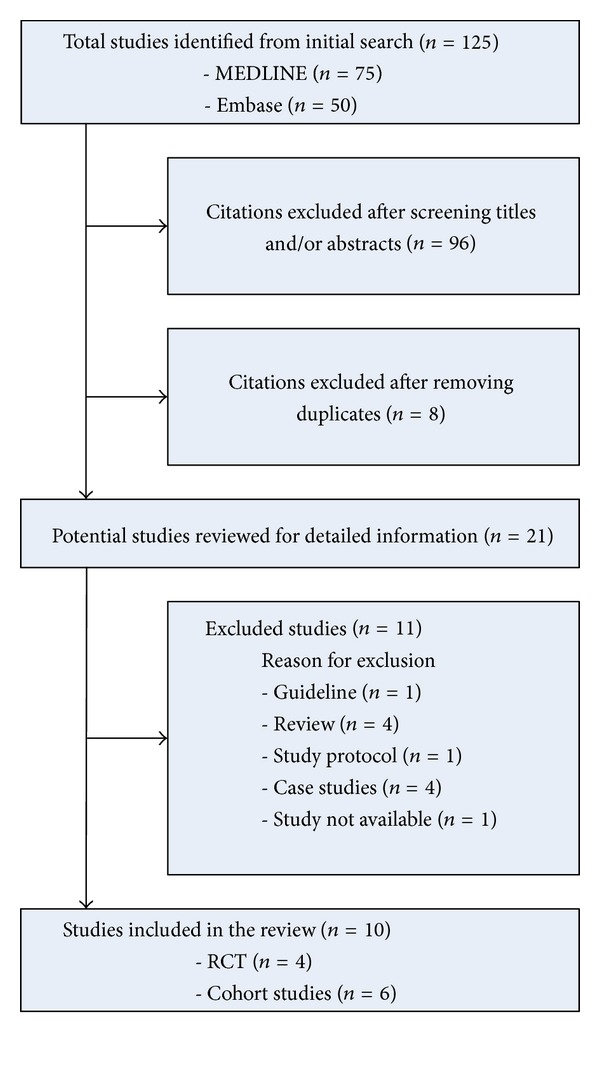
Flowchart.

The Ovid MEDLINE search (inception to November 2012) retrieved 75 records, whereas the Embase search revealed another 50 records. In total, 104 papers were excluded based on titles and abstracts or due to duplicates. Furthermore, we excluded 11 studies for other reasons: guidelines (*n* = 1), reviews (*n* = 4), study protocol (*n* = 1), case reports (*n* = 4), and study not available (*n* = 1), leaving 10 studies for inclusion in this systematic review (Figure 1). Study characteristics and results for cohort studies and randomized controlled trials are summarized (Tables 1 and 2). 

## 4. History

The first publications on the cervical pessary were small case studies in which well-defined in- and exclusion criteria as well as specified outcomes were lacking. In 1959, Cross was the first to publish on the use of the cervical pessary in 13 women with a history of incompetent cervix. Eight (62%) pregnancies went to full-term, one ended in a miscarriage, in one pregnancy an additional cervical cerclage was placed, and three pregnancies were on-going at the time of publication [10]. In 1961, Vitsky used a Smith pessary to prevent preterm birth in three patients with an incompetent cervix or a history of late miscarriage. Before treatment, these women together had six failed pregnancies prior to 20 weeks and four fetal losses between 24 and 28 weeks. In these three women, five pregnancies were treated with a pessary, of which four continued to full-term pregnancies [6].

In 1966, Oster and Javert published on the Hodge pessary as an alternative for the possibly hazardous cervical cerclage. They performed a study on women with an obstetric history of high fetal mortality rate because of recurrent miscarriage and preterm birth due to the incompetent cervical os. Before the treatment of these 29 women (94 pregnancies), 94 infants were born of whom 16 (17%) were born after 37 weeks of gestation. In the 35 subsequent pregnancies, these women were treated with a pessary and had 23 (66%) term births [8].

### 4.1. Cohort Studies

Quaas et al. treated 59 women for prophylactic and 44 women for therapeutic indications with an Arabin pessary. Reasons for prophylactic treatment were a history of miscarriages or preterm birth and multiple pregnancies. Women with a cervical conization, cervical tear, or cervical ripening (bishop score > 6) were treated with a pessary for therapeutic indications. Another five women were treated with the pessary instead of an emergency cerclage because of cervical dilation or prolapsed membranes. Four (80%) of these women had an uncomplicated prolongation of their pregnancy. In total, 98 (92%) of the patients treated with the pessary delivered after 36 weeks of gestation [11].

Arabin et al. treated 11 women with a short cervical length (<15 mm), four women with a singleton pregnancy, and seven women with a twin pregnancy, with a cervical pessary. The mean gestational age for women with a singleton was 35^+3^ and for women with a twin pregnancy 35 weeks. No one delivered before 32 weeks of gestation. Furthermore, a retrospective matched pair analysis was performed. Patients treated with a pessary were matched to patients without treatment, where cervical length did not differ more than 2 mm at the same gestational age. The mean gestation age was 38 weeks for singletons in the treatment group (*n* = 12) and 33^+4^ in the control group (*n* = 12) (*P* = 0.02). For twin pregnancies, the mean gestational age was 35^+6^ in the pessary group (*n* = 23) and 33^+2^ in the control group (*n* = 23) (*P* = 0.02). In singleton pregnancies, no one delivered before 36 weeks in the treatment group compared to six cases in the control group (*P* < 0.001). This effect was not significantly shown for twin pregnancies (8 women (35%) in the pessary group versus 12 (52%) in the control group) [12].

Antczak-Judycka et al. studied the effectiveness of the pessary (*N* = 35) versus the McDonald cerclage (*N* = 22) in women with clinically and ultrasonographically confirmed shortening of the cervix between 22–27 weeks of gestation. There was no difference in prolongation gestational age (13.4 weeks versus 12.1 weeks for cerclage and pessary, resp. (*P* = 0.06)). They showed that the choice of the method does not affect the mode of delivery as well as neonatal outcome [13].

In a prospective cohort study by Acharya et al., 32 women with a cervical length <25 mm before 30 weeks of gestation were treated with an Arabin pessary. There were 21 women with a singleton, nine with a twin, and two with a triplet pregnancy. Three women were excluded from analysis: two required early delivery due to severe intrauterine growth restriction and one due to HELLP syndrome. Delivery before 28 weeks occurred in six (20.7%) women and before 34 weeks in 13 (45%) women. The mean gestational age at delivery was 34 weeks. Neonatal outcome demonstrated the following: mean birth weight was 2,255 g, mean Apgar score at 5 min was 8 and 4 (13,8%) perinatal deaths [14].

Women with a singleton pregnancy and a cervical length between 15–30 mm before 28 weeks of gestation were treated with a pessary in a study by Sieroszewski and coworkers. Nine (16.7%) women delivered before 37 weeks of gestation. The mean gestational age at delivery was 35.3 ± 4.4 weeks. Two (3.9%) children were admitted to the neonatal intensive care unit (NICU), there were no neonatal deaths [15].

In 2010, Kimber-Trojnar et al. used a cervical pessary in 56 women with increased risk of preterm birth (such as history of miscarriages, prior PTB <34 weeks, cervical suture in previous pregnancy, history of cervical laceration or cervical coniztation, and twin pregnancies). The results demonstrate two (3.6%) deliveries before 34 weeks and eight (15%) before 37 weeks of gestation. The mean gestational age at delivery was 38.3 (30.4–41). All born infants, 58 (100%), were alive [16].

### 4.2. Randomized Controlled Trials

Among the four randomized studies, two were truly randomized while two others used quasi randomization. In 1986, Forster et al. compared 112 patients with a singleton pregnancy treated with cervical cerclage to 130 patients with a Stütz pessary. There was a third group of bedrest, but all these patients needed additional therapy, so data on this group was not reported by Forster et al. Entry criteria were not well described, and the authors used quasi randomization based on the initial of the woman's surname. Delivery before 28 weeks (0.9% cerclage versus 0% pessary group), between 28 and 30 weeks (2.7% versus 2.3%), between 31 and 33 weeks (5.6% versus 3.1%), between 34 and 36 weeks (14.3% versus 14.6%), and after 37 weeks (76.8% versus 80%) did not differ between both groups. Furthermore, there was no significant different in gestational age (37.57 weeks versus 38.15 weeks for cerclage versus pessary), perinatal mortality (2 (1.7%) versus 0 (0%)), birthweigth (3062.7 g versus 3097.9 g), or 5 minutes Apgar scores (8.62 versus 8.67) [17].

In 1991, Gmoser et al. performed a randomized prospective study in which women were assigned to a pessary group (*n* = 169) and a control group (*n* = 131). The randomization method and in- and exclusion criteria were not well described. Women in this study were at high risk for preterm birth due to cervical dilatation after contractions (*N* = 109), cervical widening without contractions (*N* = 95), a history of cervical incompetence (*N* = 18), twin pregnancy (*N* = 52), and a preterm birth despite a prophylactic cerclage in a previous pregnancy (*N* = 26). The median gestational age at delivery in the pessary group was 39 weeks versus 36 weeks in the control group. Birthweight was 2950 g in the pessary group and 2400 g in the control group [18]. 

The PECEP study was a multicenter RCT that randomized 385 women with a singleton pregnancy and a short CL (<25 mm) at routine second trimester ultrasonography (18–22 weeks) for a pessary (*N* = 192) or expectant management (*N* = 193). Five women were lost to followup. Women with a major fetal abnormality, painful regular uterine contractions, active vaginal bleeding, ruptured membranes, placenta praevia, and a history of cone biopsy or cervical cerclage in situ were not included. The primary outcome, that is, delivery before 34 weeks of gestation, occurred less in the pessary group compared to the expectant management group (6% versus 27%, RR 0.24 CI 95% 0.13–0.43), as did delivery before 37 weeks of gestation (41 (22%) versus 113 (59%) women in the pessary and control group (RR 0.36; CI 95% 0.27–0.49)), and delivery before 28 weeks of gestation (4 (2%) women in the pessary versus 16 (8%) in the control group (RR 0.25; CI 95% 0.09–0.73)). The mean gestation age at delivery was 37.7 weeks in the pessary group versus 34.9 in the expectant management group (*P* < 0.0001) [19].

Hui et al. randomized 108 women with a singleton pregnancy and a cervical length <25 mm at routine second trimester ultrasonography (20–24 weeks) to the pessary (*N* = 53) and the control (*N* = 55) group. Women with a major fetal abnormality, history of cervical incompetence, surgical cerclage in current or previous pregnancy, multiple gestation, presence of cervical dilatation, painful uterine contractions, or ruptured membranes were not included. The investigators did an attempt to blind the patients by performing a vaginal examination and simulate the insertion of a pessary in all women. The primary outcome, that is, delivery before 34 weeks of gestation, occurred in five (9%) women in the pessary group versus three (6%) in the control group (RR 1.7; 95% CI 0.43–6.9). Delivery before 37 weeks of gestation occurred in eight (15%) women in the pessary group and ten (18%) women in the control group (RR 0.83; CI 95% 0.35–1.94) and delivery before 28 weeks of gestation in two (4%) women in the pessary versus three (6%) women in the control group (RR 0.69; CI 95% 0.12–3.97). The mean gestation age at delivery was 38.1 weeks in the pessary group versus 37.8 weeks in the expectant management group [20].

## 5. Discussion

This systematic review included six cohort studies and four randomized controlled trials studying the effectiveness of a cervical pessary to prevent preterm birth. The cohort studies indicated potential effectiveness of the pessary, while the randomized studies showed conflicting results.

The PECEP trial demonstrated a significant reduction of preterm delivery of gestation by treatment with a pessary, but this effect was not confirmed by Hui et al. [19, 20]. A possible explanation for the difference between these studies might be the fact that the preterm delivery rate was much higher in the control group from the PECEP trial (8% before 28 weeks, 27% before 34 weeks and 59% before 37 weeks) than in the study by Hui et al. (6% before 28 weeks, 6% before 34 weeks, and 18% before 37 weeks). A study by Blencowe et al. for worldwide estimates of preterm birth rates demonstrated similar results for China and Spain (7.1 versus 7.4, resp.) [21]. Although women with a short cervical length have a 3–6 folds increased risk, this does not clarify the variance between both studies [22, 23].

Differences in the baseline preterm birth rate are partially explained by differences in baseline characteristics between the two studies. On baseline, the PECEP study population had a higher BMI (24.7 versus 21.8), and had a higher prevalence of smoking (19.5% versus 3.7%), had more women with a history of preterm delivery (11% versus 8.3%), and the population was mostly white or Latin American. Nevertheless, further evaluation is required to clarify whether the PECEP study recruited women with additional risk factors that could explain the differences between preterm delivery rates. 

Furthermore, both studies demonstrate differences in their sample size calculation. The PECEP needed to recruit 380 women in five hospitals to show a reduction of PTB <34 weeks from 28% to 14% [19]. Hui et al. recruited in one centre with a target sample size of 1120 women to demonstrate a reduction of PTB before 34 week from 8% to 4% [20]. Enrolment was slow with 100 women recruited in 29 months. To determine whether to reevaluate the sample size or extend the study to other centers, the authors decided to analyse their data for the first 108 women. 

The rates of PTB <34 weeks were lower than expected in the control group (actual 5.5% versus expected 8%). Around the same time, the results of the PECEP trial were published demonstrating different outcomes. Hui et al. decided to stop their study and publish their results. Since this study did not achieve their target sample size and, therefore, most likely is underpowered, it is difficult to formulate recommendations for clinical practise based on these results. 

Although, due to the nature of the intervention it was impossible to blind randomization. Hui et al. did an attempt to blind the patient to the allocation. Patients in the control group received a vaginal digital examination simulating the insertion of a pessary. Treatment allocation was only revealed to the obstetrician in charge after the patient was consented. The use of a pessary might have affected medical decision making, but we believe that the fact that patients were blinded does not explain the different outcomes between both studies.

The safety of the treatment with a pessary is investigated in several studies. Overall, the insertion and removal of the pessary is simple and well tolerated by women. Arabin showed, in a patient prevalence questionnaire evaluation, that 75% of the treatment group would use the pessary again and would even recommend it to others. They reported that there might be some increase of vaginal discharge [12]. According to the maternal satisfaction questionnaire used in the PECEP trial, 95% of women would recommend the vaginal pessary to other people [19]. A study in 200 pregnant women with a pessary was compared to women with normal pregnancies concerning microbiological findings and puerperal morbidity. There was no higher infection morbidity compared to the control group [24].

Despite our extensive search, we could only identify four randomized controlled trials, of which two fulfilled the current standards. It is remarkable that an intervention used since 1959 has been evaluated in several prospective cohort studies or non-randomized comparative studies and only in two well-designed RCTs. 

Recently, Chang et al. reported an analysis of trends and potential reductions with interventions in countries with a very high human development index. They formulated a target of a 5% relative reduction of preterm birth rate from 9.59% to 9.07% of live births, using smoking cessation (0.01 rate reduction), decreasing multiple embryo transfers during assisted reproductive technologies (0.06), cervical cerclage (0.15), progesterone supplementation (0.01), and reduction of nonmedically indicated labour induction or caesarean delivery (0.29) [25]. The use of a pessary was not used in their recommendations, obviously due to the limited evidence on the subject.

The studies mainly included women with a singleton pregnancy at high risk for a preterm delivery. Women with a multiple pregnancy are at increased risk for preterm delivery too. In The Netherlands, approximately 50% of women with a multiple pregnancy deliver before 37 weeks of gestation, of whom 9% even before 32 weeks [26]. In the United States, these rates are 60% and 12%, respectively. In comparison, in women with singleton pregnancy 6%–10% deliver before 37 weeks and 1% before 32 weeks. Future research should also focus on the use of a cervical pessary in multiple pregnancies [27].

In conclusion, the cervical pessary seems an affordable, safe, and reliable alternative for prevention of PTB in a population of appropriately selected at-risk pregnant women who have been screened for cervical length assessment at the midtrimester scan. In view of the differences in outcomes between Hui et al. and the PECEP trial, further research is urgently needed to confirm the efficacy of cerclage pessary in prevention of preterm birth.

## Figures and Tables

**Table 1:** Prospective cohort studies.

Author	Year	Country	No. of women	Inclusion criteria	Exclusion criteria	Age	Type of pessary	Pregnancy outcome	Neonatal outcome	Pessary removal and side effects
Quaas	1990	Germany	107	Women with history of miscarriages Preterm birth, multiple pregnancies, Cervical conization, cervical tear, cervical Ripening (bishop score >6), dilation or Prolapsed membranes	Not clearly stated	Not stated	Arabin	92% of women treated with pessary delivered >36 weeks	Not stated	36 weeks of gestation

Arabin	2003	The Netherlands	11	History of spontaneous preterm birth <36 weeks and a CL <15 mm (singletons and twins)	Severe regular contractions Blood loss Premature rupture of membranes	24–43 years	Arabin	Singletons: mean GA at delivery 35 + 3 weeks Twins: mean GA at delivery 35 weeks Pessary versus control: singletons <28 weeks: 0 (0%) versus 2 (17%) <32 weeks: 0 (0%) versus 3 (25%) <36 weeks: 0 (0%) versus 6 (50%) Pessary versus control: twins <28 weeks: 0 (0%) versus 1 (4%) <32 weeks: 0 (0%) versus 7 (30%) <36 weeks: 8 (35%) versus 12 (52%)	Not stated	17 (58%) complains of discharge 13 (44%) pain during insertion 15 (52%) pain during removal 28 (97%) recommend to others

Antczak-Judycka	2003	Poland	57	Women with a risk of PTB: shortening of cervical length or dilatation between 22–27 weeks	Intrauterine infection contractions Urine tract infection Vaginal infection	20–46	Not defined	Cerclage versus pessary Prolongation of pregnancy: 13.4 (±3 weeks) versus 12.1 (±3 weeks) Mean GA at delivery: 37.3 (±2 weeks) versus 37.7 (±3 weeks) Delivery >37 weeks: 17 (77%) versus 31 (89%)	Cerclage versus pessary birthweight: 3080 ± 676 versus 3063 ± 826 Apgar score 5 min: “good” perinatal death: 2 (6%) pessary group	Cerclage versus pessary PPROM: 1 (5%) versus 2 (6%) Premature contractions: 2 (10%) versus 2 (6%) Cervical tear: 3 (15%) cerclage group Pessary dislocation: 1 (3%)

Acharya	2006	Norway	32 (21 singletons, 9 twins, 2 triplet)	Women with a riskfactor for PTB and a short CL (<25 mm) before 30 weeks of GA	No viable fetus or congenital malformations	23–39 years	Arabin	Delivery <28 weeks: 6 (20.7%) Delivery <34 weeks: 13 (45%) Mean GA at delivery 34 weeks	Mean birthweight: 2255 g Mean Apgar score at 5 min: 8 Perinatal death: 4 (14%)	34 + 36 weeks 2 removal due to pain Increased discharge in all women

Sieroszewski	2009	Poland	54	Singleton pregnancies with a CL 15–30 mm before 28 weeks	Multiple pregnancies Fetal malformations Uterine contractions PPROM Blood loss Abnormal placenta localisation	<20 years 1 (1.9%) 20–34 years 47 (87%) 35–39 years 4 (7.4%) >37 years 2 (3.7%)	Arabin	Delivery <37 weeks: 9 (16.7%) Mean GA at delivery 35.3 weeks	Apgar score scales: 0–4: 2 children (3.7%) 5–7: 7 (13%) 8–10: 45 (83.3%) NICU admission: 2 (39%) 0 neonatal deaths	Mean gestational age at removal 33.3 weeks No complications or side effects

Kimber-Trojnar	2010	Poland	56	Women with a riskfactor for PTB: history miscarriages, prior PTB <34 weeks, cervical suture in previous pregnancy, history of cervical	Contractions, ruptured membranes, maternal pyrexia, elevated CRP or white blood cell count, vaginal	19–43 years	Polyvinyl chloride pessary	Delivery <34 weeks: 2 (3.6%) Delivery 34–37 weeks: 6 (10.7%) Delivery >37 weeks: 48 (85.7%)	Mean birthweigth: 3255 Mean Apgar score at 3 min: 9, 6 58 (100%) live born infants 7 (12.5%) NICU admission	37 weeks, contractions vaginal bleeding, discomfort fetal distress, ruptured membranes

**Table 2:** Randomized controlled trials.

Author	Year	Country	No. of women	Inclusion criteria	Exclusion criteria	Age	Type of pessary	Pregnancy outcome	Neonatal outcome	Pessary removal	Side effects of pessary
Forster	1986	Germany	242	Not clearly stated	Not clearly stated	Mean age pessary: 26.6 sd 6.13 Mean age cerclage: 26.3 sd 4.22	Stützpessary	Pessary versus control mean GA at delivery: 35.15–37.57 weeks	Pessary versus control birthweight: 3097.9 versus 3062.7 g Neonatal mortality: 0 (0%) versus 1 (0.89%)	Not stated	Not stated

Gmoser	1991	Germany	300	Women with cervical changes (with or without contractions), history of cervical incompetence, twin pregnancies or a previous pregnancy with a cerclage	Not clearly stated	Not clearly stated	Stutzpessary	Pessary versus control median GA at delivery: 39–36 weeks	Pessary versus control birthweight: 2950–2400 gram	Not stated	Not stated

Goya	2012	Spain	385	Singleton pregnancies CL <25 mm at 18–22 weeks	Major fetal abnormalities Regular uterine contractions Active vaginal bleeding Ruptured membranes placenta praevia History of cone biopsy cervical Cerclage in situ	18–43 years	Arabin	Pessary versus control delivery <28 weeks: 4 (2%)–16 (8%) delivery <34 weeks: 12 (6%)–53 (28%) delivery <37 weeks: 41 (22%)–113 (59%) mean GA at delivery: 37.7–34.9 weeks	Pessary versus control composite adverse outcome: 5 (3%)–30 (16%) birthweight < 1500 g: 9 (5%)–26 (14%) birthweight < 2500 g: 17 (9%)–56 (29%)	37 weeks of gestation Risk of PTB with persistent Contractions despite tocolysis Vaginal bleeding Patient discomfort	All women had increased vaginal discharge

Hui	2012	China	108	Singleton pregnancies CL <25 mm at 18–22 weeks	Major fetal abnormalities Regular uterine contractions History cervical incompetence Ruptured membranes multiple gestation Cervical dilatation Cervical cerclage: current or previous pregnancy	22–44 years	Arabin	Pessary versus control delivery <28 weeks: 2 (3.8%)–3 (5.5%) delivery <34 weeks: 5 (9.4%)–3 (5.5%) delivery <37 weeks: 8 (15.1%)–10 (18.2%) mean GA at delivery: 38.1–37.8 weeks	Pessary versus control Clinical sepsis: 3 (5.7%)–5 (9.4%) Jaundice: 15 (28.3%)–14 (26.4%) RDS: 5 (9.4%)–2 (3.8%) IVH: 0 (0%)–1 (1.9%) NICU admission: 21 (39.6%)–17 (32.1%)	37 weeks of gestation Ruptured membranes Vaginal bleeding Painful contractions	25 (47%) increased discharge 4 (8%) pressure sensations 2 (4%) dislodge pessary 1 (2%) vaginal bleeding 1 (2%) pain 1 (2%) urine retention
